# A Research on Online Education Behavior and Strategy in University

**DOI:** 10.3389/fpsyg.2022.767925

**Published:** 2022-04-25

**Authors:** Quan Deng

**Affiliations:** School of Art and Communications, Guangzhou College of Applied Science and Technology, Guangzhou, China

**Keywords:** deep learning, artificial intelligence, educational strategy, university adaptive learning, educational behavior

## Abstract

After the reform and opening up in China, through a series of rapid developments in world, online education has grown both socially and economically. This area has become representative of the fast-growing economy. However, Guangfu culture as a crucial component of Cantonese traditional culture is gradually becoming less influential today. It is the college's responsibility and duty to protect, carry forward, and inherit this traditional culture. Especially during this cyber era, where networks have become a powerful source for communication and study, there are diversified methods of adaptive learning and various learning behaviors. This article aims to analyze the plausibility of adapting an online platform into the teaching arena and the needs of students under this teaching mode. A simulation of applying advanced technology and artificial intelligence into Guangfu culture innovation was also conducted. The contribution shows the users in this platform have a longer study time, compared with non-platform users, and are more interested in traditional culture knowledge than non-users; 21.5% higher in the performance's test.

## Introduction

The Internet era has come, and according to the jargon of the computer industry, it is also known as the “WEB2.0” era. Today, there are too many cases to prove the Internet's saturation in modern society. The most obvious evidence of the Internet's popularity can be seen in a government report from 5 March 2015. At the 12th session, Premier Li mentioned the “Internet+” initiative, which represents the beginning of a new kind of economy. The “Internet +” strategy involves almost every aspect, including study, work, entertainment, and economy. With a few clicks, you can take part in a series of lessons, work, entertainment, or any other activities through the Internet. The revival of traditional folk culture has also been accelerated. Education integrated with Guangfu culture benefits a lot from all kinds of excellent online education websites (Cao et al., 2022; Han et al., 2022). The faster the learning process, the more publicity it will get. Even though this model lacks details, it does provide the basis for Guangfu culture's revival. The outbreak of COVID-19 accelerated the deep learning of the cyber era, it has become an unavoidable trend for this generation. In Park, as long as you use a mobile phone to scan the official QR code, the “five sheep” sculpture story explanation course will present in front of you immediately. The successful publication of the Yuexiu government showed us the possibility to team up the dissemination of Guangfu culture with the Internet (Huang et al., 2019; Chen et al., 2022).

In recent years, our country's electronic industry has been developing rapidly, many aspects of technology are on the cutting edge. However, with the fast growth of the Internet, we sometimes get caught in confusion. To keep up or to give up has become a choice for everyone. The development of the Internet has also met a bottleneck, not for technical factors, but for problems emerging in cultural identity. At this particular moment, we need a way out. The “Internet +” culture communication might be the solution. But how far can it go? From the author's perspective, knowledge sharing and education development are crucial for human civilization. Therefore, no matter how many years have passed or how many changes are made in the education mode, education and cultural inheritance remains to be the essence and treasure of the Chinese nation. As long as we observe carefully, we will find that the traditional culture is affecting every aspect of our life, especially the Guangfu culture to Cantonese (Tegen et al., 2021; Abdelkader et al., 2022). These traditional cultures are actually the sources of our cultural identity. However, today's younger generation, under the impact of the current popular culture, are gradually moving away from the traditional culture, which is why they are feeling a sense of lost. Integrating fashionable “Internet + with the Guangfu culture” might be able to tackle the problem and create a new symbol of modern fashion integrated with traditional culture (Huang et al., 2017; Chen et al., 2021c).

The Pearl River Delta region is the birthplace of Guangfu culture. Guangfu culture is a subordinate and an important representative of Lingnan culture, which arose from the “Ren Qicheng” era, more than 2,000 years ago. The emergence of Guangfu culture has made great contributions to the social, political, economic, and other aspects of the Pearl River Delta region. Guangzhou is located in the center of the Pearl River Delta. Ever since ancient times, it has been an important “port” for culture communication. Due to the reform and opening up in China, life standards in the Pearl River Delta area have gradually elevated while the stress of life has increased. As the pace of urban life is getting faster and faster, people's attention on Guangfu culture and tradition is gradually fading. To further illustrate this phenomenon, we should take a closer look at the following statements: young people under 30 have no deep understanding of Guangfu culture. People over 35 have a deep attachment to Guangfu culture. With the revival of Guangfu culture, the ratio of Cantonese-speaking pupils is decreasing. Nearly half of “New Guangfu people” do not have a clear vision of the significance of inheriting the Guangfu culture (Abbas et al., 2015; Yan et al., 2016).

Apart from that, the language barrier is also an inducement to hinder the development of Guangfu culture. Most of the “new Guangfu people” can only obtain Guangfu culture-related information from traditional media such as TV or newspapers, most of which is written in Cantonese. Different from Mandarin, most of these materials have a very unique culture system. Putting language aside, the propaganda of Guangfu culture is relatively widespread, there are very few people who know how to obtain relevant information about Guangfu culture through the Internet, which is also an incentive for blocked cultural transmission. As students' needs for the Internet are rising, this makes them powerful transmitters for Guangfu culture. Currently, websites with rich resources and well-designed interfaces are most eye-catching. Promotion of Guangfu culture through traditional media, such as articles or single lectures, is likely to be fruitless. Even though Guangfu culture has already formed a specific concept and research mode in lectures or articles that have contributed greatly, these kinds of research articles have failed to achieve the goal of “elegant and vulgar common appreciation” and “stick to the public,” which greatly impacts the publicity of Guangfu culture. All the phenomena mentioned above show that in the combination of the “popular and tractional,” the integration of Guangfu culture and online education is imperative.

At present, both at home and abroad, there is a fierce debate on online education and on traditional culture research, but most of the research is structural, mainly conducted on education technology, education methods, and so on, but almost none have combined educational behavior with education strategy. By collecting and re-sorting the information, we found that although traditional culture research has taken up a large proportion in the research field, most of the studies are mainly about teaching techniques, teaching methods, and teaching resources, etc. Due to the uniqueness of Guangfu culture, there is no relevant article at the moment. Innovatively, this article will analyze the development and inheritance of traditional local culture from the perspective of online education, and explore the value and significance of integration of local culture and online education.

Some foreign scholars have made great achievements in the study of online education systems, and some of the concepts and practices of online education have been applied in students' daily study. In 2012, some well-known universities in the United States adapted online education and achieved good results. After that, many other universities designed their own online learning and teaching platform. Students have shown great interest in online courses. Therefore, this kind of new education method has been adapted by more and more universities, especially under the pressure of the epidemic. This kind of teaching model also shows prominent commercial value, as more and more online course suppliers are emerging, such as Coursera and Udacity. By choosing good teachers and high-quality courses, these suppliers provide many excellent education programs for their audiences. “Home schooling” is no longer viewed as other, it has become the trend of most university courses. While online education is receiving all the praise in the United States, many overseas countries are also beginning to adopt online education courses. Coupled with overseas curriculum suppliers, European and Asian universities have introduced an online education teaching mode. Taking Singapore for example, Coursera marketing has cooperated with the National University of Singapore to build an online platform. Taking Coursera as an example, it was originally founded by two professors from Stanford University in 2013, and since then has garnered over 1.5 million course applicants, 680,000 registered students, registered as many as 124 courses, and the company's market value has reached $65 million.

The advanced development of MOOC's artificial intelligence has been proven in foreign countries, which is also the reason for the quick uptake of the domestic online education market. In 2013, EDX, an American online education platform, reached out to Tsinghua University and cooperated with them, making it the first Asian university partner, increasing EDX's influence in China. During the cooperation with EDX, Tsinghua University introduced a lot of excellent teachers and high-level teaching teams to establish MOOC groups, and most of the excellent courses are open to the whole world. In the same year, two other famous universities, Shanghai Jiao Tong University and Fudan University announced their cooperation with another “MOOC” port Coursera in the United States to further develop online course. From 2013 to 2015, many domestic universities cooperated with an MOOC platform in the United States. Online education behavior is favored by domestic universities and attracted the attention of the Ministry of Education. In 2014, the Ministry of Education issued the “national boutique open course initiative,” a famous domestic network enterprise called NetEase, and proposed a “cloud classroom” to promote the Chinese MOOC of independent domestic property. Meanwhile, “Love course” also launched under the “China University MOOC” project. Today, the “MOOC” project is favored by many domestic students. Simple operation and diverse interactions attract more and more college students to make use of MOOC learning. China has started MOOC from an education behavior aspect to develop education strategy, artificial intelligence, and public deep learning.

## Related Work

### Guangfu Culture

There are three general characteristics of Guangfu culture: shared, intersective, and open. These characteristics are greatly related to the geographical location of the Guangdong region. Guangfu culture is a shared culture. Ever since the six counties were unified, benefitting from its unique coastal geographical location and diverse climate, this region has been enjoying rich nature resources. After that Zhao Tuo, the administrator, came to the county, and brought a large number of populations to this area. Although friction came along with the immigration, after a period of integration, the “symbiosis” thought from the ancient Chinese mainland harmonized the Han people and the local south Vietnamese people and they finally reach an agreement to share resources. After that, under the sharing cultural atmosphere, the Pearl River culture integrated with the immigrant culture, slowly transforming into new kinds of distinctive characteristics in Guangfu culture (Chen et al., 2021a,b).

Guangfu culture is an intersective culture. Historically, most of the Lingnan region's population are immigrants, which means this region has conducted large-scale cultural exchanges and received most of the advanced consciousness and production technology from the north. In several major waves of migration of Chinese history, Guangdong is an important destination for immigrants. Many areas of Lingnan were unexploited due to the severe natural environment, and therefore natural resource storage was very rich. This also benefited the “new Lingnan” people due to “gold mining.” As the “New Lingnan people” came from all over the Central Plains, all the local cultural elements converged here. The blending of all kinds of cultures further enriched the content of Guangfu culture.

### Characteristics of Online Education Behavior

To simplify the concept, online education is also a new form of education, it can be regarded as a kind of distance education rely on electronic devices. To carry out online education, there must be equipment that enables Internet access as the classroom or learning method is highly digitalized. It is a new education behavior under this new era. Its form is highly digitalized, coupled with complete network interactive characteristics (Huang et al., 2015; Chen et al., 2020a). It can be characterized as the following:

1. Open nature: The first nature of online education is openness, it is the basic characteristic of online educational behavior. The base of traditional education is in school, school education methods and learning resources are limited and very few universities own the ability to serve as a scientific research sample. There is no doubt that once online education becomes universal, it will serve all classes of society. That is to say, primary school students can also learn college context by simply opening a courses website to be taught a certain theory. This is true zero barrier era for all learners. In addition, the educated population will also increase. But the scarcity of teaching resources and costs are deteriorating, educational resources and information will become a scarce resource that will have to be shared. In 1999, the Ministry of Education initiated the Action Plan for Education Revitalization of the 21st Century for HP National Education. Even though the number of universities has tripled in 13 years, The Opinions on Comprehensively Improving the Quality of Higher Education issued in 6 April 2020 by the Ministry of Higher Education also reflects the shortage of university resources that might increase the education budget. However, if online education is popularized, then universal study will be become people-centered again. The purpose of online education is to let everyone have an equal learning opportunity and right.

2. Extendibility: The extendibility of online education is a functional characteristic. Those well-known universities commonly practice recruitment from the best high schools; this kind of practice can even begin in middle school. These schools collect all the good educational resources, integrate a good teaching mode, concentrate on education and the best students to achieve good results, and get their students admitted to their ideal university. But this kind of model only benefits a small number of talented students, only a few students can enjoy quality teaching services. On the other hand, online education can fulfill in-time communication, online interaction and take advantage of other technologies, and transfer high-quality educational resources to under-developed areas and all around the world. It can provide learners with better and richer choices (Huang et al., 2012; Chen et al., 2021b). The top universities and the best middle schools will not waste educational resources and financial allocation as these education resources can be shared online, anytime and anywhere. This extension of education reflects the concept of modern education, online education, as a new idea of education development.

3. Flexibility: As mentioned above: “anytime and anywhere.” Flexibility is another important characteristic of online education. The Internet is developing rapidly. Fifteen years ago, it was not very popular, the content was plain and transmit paths were relatively single, even though online education could be conduct by that time, it was quite boring. But today, as long as you process a mobile phone, you can learn anytime and anywhere with joy. By using the “cloud technology” and large databases, online education is given a richer, more flexible content and meaning. Online education will slowly overthrow traditional education. To break the barrier of a university, introducing a more flexible trend to the enrollment mode management, school status management, talent training program, learning mode, and curriculum mode is beneficial.

4. Intermediation: The learning characteristic of online education is communication, which is intermediary. Online education learning often relies on Internet technology, communication technology, etc. There are various high-quality educational resources which are yet to be put online. With the development of “cloud” technology and with the help of new technology, such as artificial intelligence, long-distance education projects might be able to be carried out. Therefore, today, each node in modern online education, such as registration, teaching activities, homework assignment and submission, and online interaction between teachers and students are actually inseparable from the intermediary role of the Internet (Liu et al., 2014; Lin et al., 2020). The traditional form of school education might also apply electronic technology, but it is more independent than online education when it comes to Internet reliance. So, without the intermediary role of Internet technology, online education will be void.

5. Manageability: Management ability is indispensable to online education. After years of study, there is a set mode of interpersonal management, but how to manage the openness, extensiveness, flexibility, and mediation of online education remains a question. This problem gives management research a new challenge. Online education is a new education behavior mode. Therefore, the management of online education must be suitable for all the characteristics mentioned above.

### Local Culture and Education Strategies in Colleges and Universities

Local traditional culture is an important part of traditional Chinese culture, it has unique cultural complicity and ideological feelings to precious cultural heritage. The key to carry traditional culture forward is to dig deeper, sort out, develop in practice, and innovate in inheritance. The new era gives colleges and universities a new historical mission, and we must undertake the important responsibility to carry that forward, to lead and to inherit the local traditional culture. University is an important subject in carrying forward Guangfu culture. As a cultural institution, one of the functions of universities is to choose and criticize, inherit, and innovate traditional culture, so as to realize the goal of cultural education and leading advanced culture. In any era, the culture of any country is its essence. The inheritance of culture by colleges and universities does include choice, colleges must inherit and carry forward the traditional culture selectively (Chen et al., 2020b, 2022). Higher educational institutions need to choose and inherit advanced and conceptual value content according to their own value trend and value concept. These institutions inherit and carry forward the essence of the times and ethnic culture. Local universities should critically choose local traditional culture, selection and criticism are the promises for local universities to inherit and carry forward local traditional culture.

(1) To build up the leading role: Colleges and universities play leading roles in promoting the Guangfu culture. What they need to do is manifested in the excavation, mining, and refining of the spirit of Guangfu culture, reshaping of the cultural characteristics, and the pursuit of cultural harmonious value in the new era. To enhance the leading role of local colleges and universities, we should start by cultivating school cultural confidence. First of all, we need to improve the cultural confidence of school leaders. Only through the comprehension and consideration at the leadership level can we establish an institutional guarantee, a long-term mechanism of university cultural confidence, and cultural consciousness of Guangfu culture to ultimately serve local economic and cultural development. Secondly, we need to improve the cultural confidence of teachers. Teachers play a crucial role in inheritance, continuation, and innovation of Guangfu culture education. Teachers must take culture inheritance as their own responsibility, to absorb outstanding foreign culture to help with the development of Guangfu culture, cultivate students' cultural quality and cultural realm (Ye et al., 2019, 2021). Integrating online education with Guangfu culture is necessary. Third, enhancing students' cultural confidence is vital. We need to develop students' appreciation of Guangfu culture, guide students to analyze, conduct concept guidance, and correct behavior methods. Cultivating students' common value of culture inheritance is important, so that college students can form a higher degree of cultural confidence on the basis of a shared cultural resource (Zhang et al., 2013; Zhou, 2014).

(2) To formulate a feasible target system: A scientific target system is a necessary strategy for universities to promote Guangfu culture. To carry forward and inherit the Guangfu culture, universities should establish the basic goal of establishing and promoting the Guangfu cultural management mechanism, improving the infrastructure of Guangfu cultural displays, and elevating the cultural sense of teachers and students. We need to establish an institutional guarantee system with the joint participation of the whole school, and make it a consensus and unified action. In a healthy cultural environment, we should be able to absorb the nourishment of Guangfu culture, and to achieve the goal of a strong cultural environment (Fu et al., 2022).

(3) To build a well-oiled mechanism: Building a well-oiled mechanism is the promise for universities to carry forward local traditional culture. First of all, the guidance mechanism should be established, guided from the leadership system and in the ideology aspect, reflected in the school orientation, talent training, study style construction, school style construction, training discipline, construction, etc. It should be guaranteed thanks to the management mode and team structure. Secondly, we should establish an interactive mechanism and adhere to the concept of cultural education. Based on the integration and interaction of university cultural construction, coordinating development direction, strengthening the interaction and integration of different majors, and promoting coordination and reference between disciplines to realize the cultural construction and development of universities in the form of cultural interaction assisted by each other is vital (Zwicky and Vogel, 2006).

(4) Improving the overall cultural quality of the school: Unique cultural literacy is the core competitiveness of universities. The cultural accomplishment of colleges and universities come from cultural construction. Inheriting and carrying forward traditional culture is the cornerstone of university cultural construction. Advocating academic quality is the value of high school cultural quality. To carry forward the Guangfu culture, we should adhere to a rational and independent academic thinking pattern and study. Having school management helps academic services improve the cultural quality of local universities. First of all, we should establish the university's spirit of seeking truth and rational criticism, and cultivate a cultural and ecologically friendly environment with the university spirit as the core. Then this cultural and ecologically friendly atmosphere can permeate into every aspect of university cultural construction. Secondly, we should reflect the orientation and philosophy of the university spirit and ideology pursuit, standardize the quality and characteristics of talent training, guide the direction of school development, and improve the quality of running schools (Chen et al., 2012; Bibi et al., 2016).

## Analysis of Online Education of Guangfu Education

Any website's development, before entering the coding stage, needs to analyze the demand of their target client. Demand analyzing work plays a very important role in the software development process. To code, there must be a clear vision of the website's functions and services attractive to the website users. These are the premise of developing a success website. The demand analyzing of the Guangfu Culture Online Education platform started from the NetEase cloud classroom, a relatively successful online education platform, which conducted a detailed demand analysis by listing its advantages and disadvantages. Today, the online education industry is developing rapidly in China. There are a lot of widely known online education platforms arising in China. These platforms also attract millions of learners to learn through various forms and each platform has different learning priorities. In the initial phase, the Chinese online education industry mostly referred to the existing successful cases of the United States. But in recent years, education platform development with distinguished Chinese characteristics has gradually emerged. The technical methods and curriculum forms are diversified. For example, live broadcast, video recording, test question library, course online comment, courseware download, interactive question answering, questionnaire survey, and tracking learning can all be forms of online learning. In the research process, this article analyzes the “NetEase Cloud Classroom,” a representative of online education platforms in China.

### Analysis of the Existing Online Education Platform

**Figure 1:**
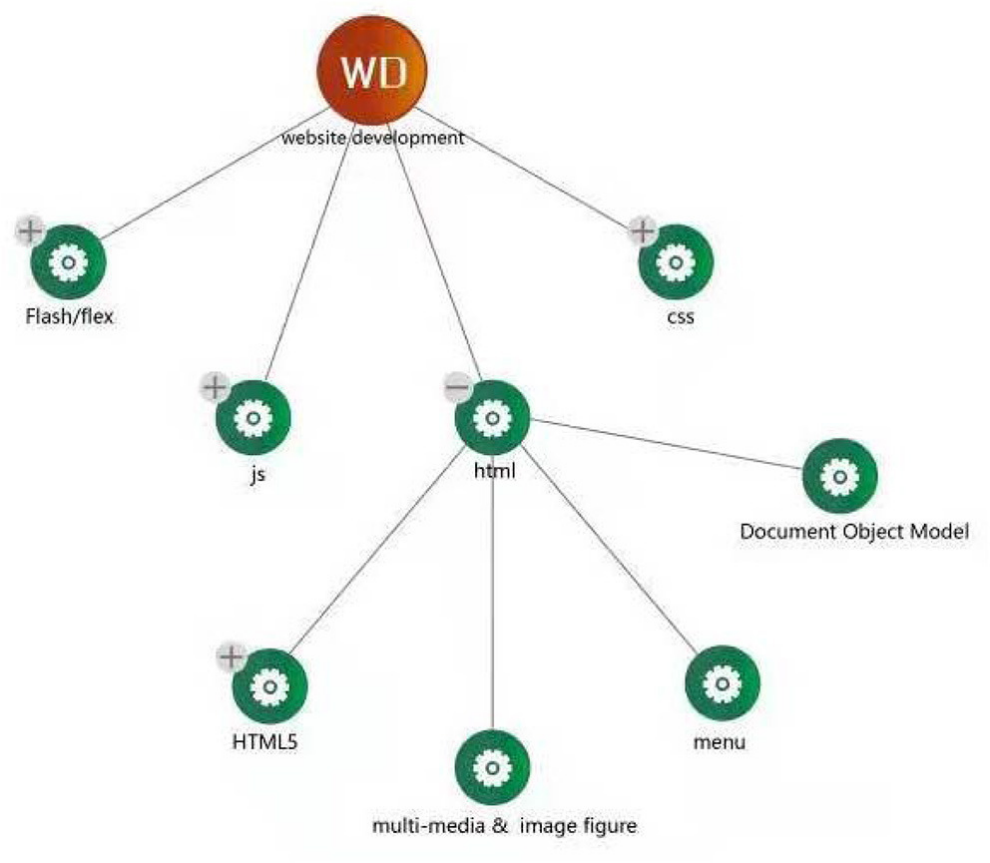
Demonstration for website development.

In 2012, the “NetEase cloud” classroom platform was established, which was relatively earlier than its competitors. It mainly provides independent network teaching services. According to the NetEase cloud classroom introduction in their “about our” pages: No matter which crowd you belongs to, no matter if you have graduated from university or are just starting, or if you require these kinds of skills at work, “NetEase Cloud” classroom is hoping to help whoever needs to learn. Many high-quality courses are available in “NetEase Cloud Classroom” and quality learning services are available as customers wish. Finally, their aim is to meet the core needs of learners to set and establish a learning curriculum. NetEase established “my cloud classroom,” as shown in Figure 1, offering options such as “online courses,” “completed courses,” and “collected courses.” Through these three services, learners can record their lifelong learning behavior data.

Through analyzing the existing relevant resources and platforms on the Internet, we have found that the advantages and disadvantages of online education platforms on the Internet are uneven, and the focus of each platform is different. This article has sorted out the advantages and disadvantages of the existing online education platforms, which are listed as follows: The advantages of existing online platforms are quite obvious. (1) The user experience is very smooth: The UI design of various mainstream online education platforms on the Internet is first-class, they can provide their users with a great learning experience. (2) Convenient, easy, and smooth operation. This feature should be adapted in the Guangfu cultural online education platform. Disadvantages of many online education are not outstanding. (1) There is no educational significance in many courses of mainstream education platforms, they are mere entertainment or sensationalism. (2) There is hardly any interaction between teachers and students, which during online courses, is hard for teachers or students to pay attention to. The Guangfu culture online education platform through the establishment of a BBS module can effectively solve this kind of problem.

### Division of the Guangfu Cultural Online Education Platform Module

**Figure 2:**
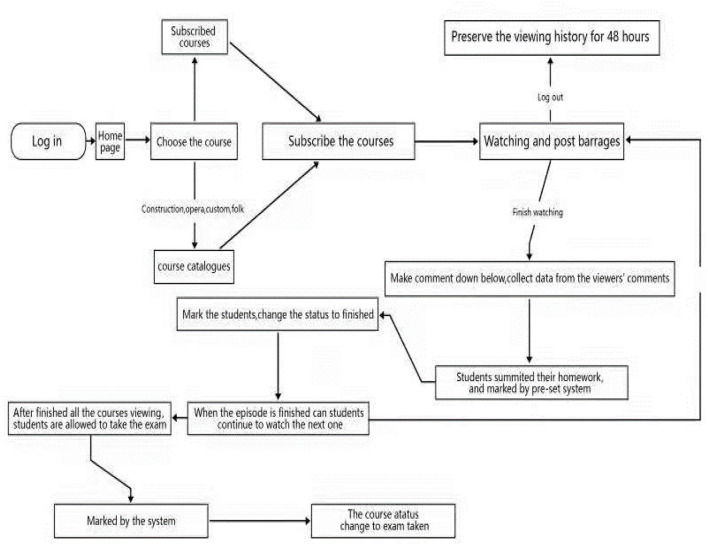
Map of the user operation flow module.

We initially divided the Guangfu cultural online education platform into three major modules, which are: The background management module, the user operation module, and the forum module. Ordinary users can only operate the user operation module, while the background management module is operated by management personnel, which allows them to use higher authority in the background management module to add, delete, and check the data sheet. The forum module is oriented to all users and can be posted and discussed in the forum. The system rights of the whole platform are divided into three kinds: Ordinary users, teachers, and administrators. The operation flow of the user operation module is shown in Figure 2.

**Figure 3:**
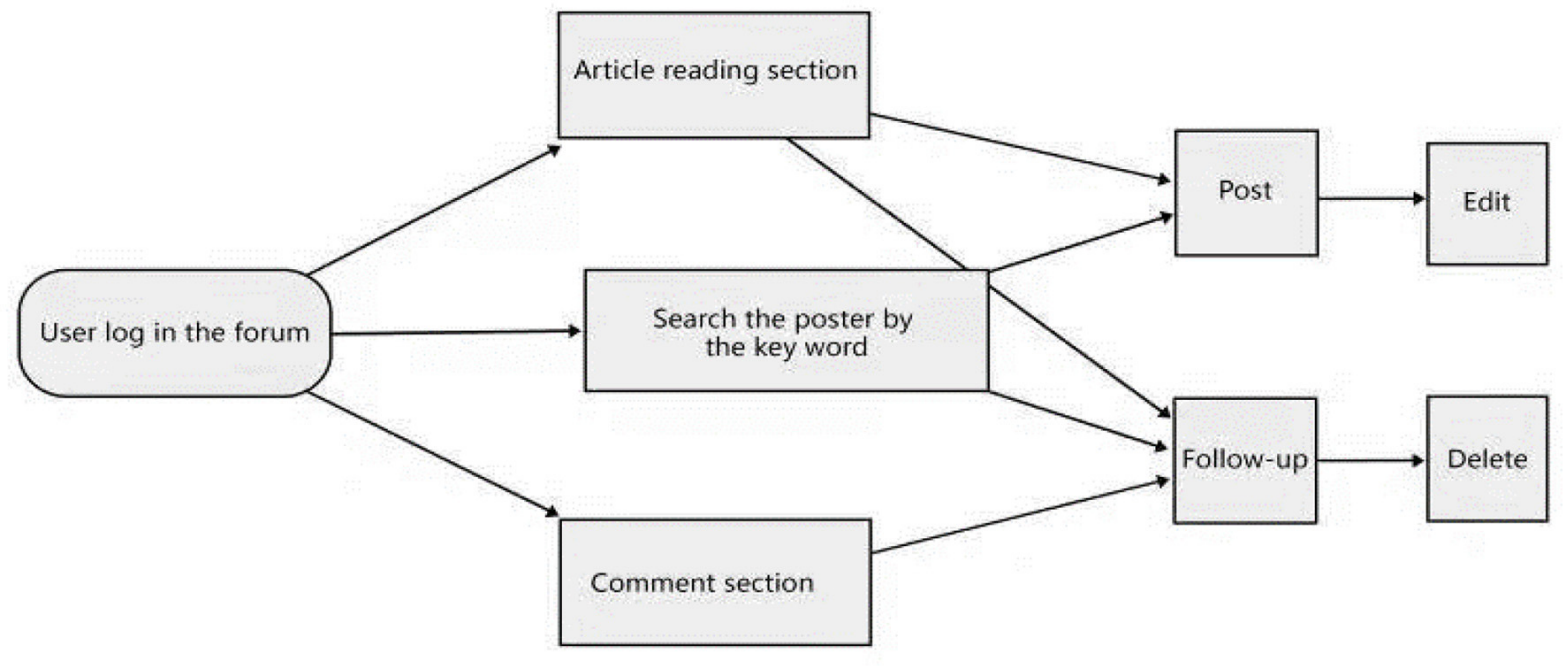
Operation flow chart of the module forum.

There are three main states: The watching process of the course, completion processing, and the completed progress. Once the user subscribes to the course, the ID of the course will be added to the user table, with information for each episode of the course. The course completion progress is divided into: Completed, ongoing, pending, and expired. Expired refers to when the user fails to complete the course within 3 months from the beginning of watching the course. The set completion progress is divided into: Completed, ongoing, and submission. The general operation flow of the forum module is shown in Figure 3.

**Figure 4:**
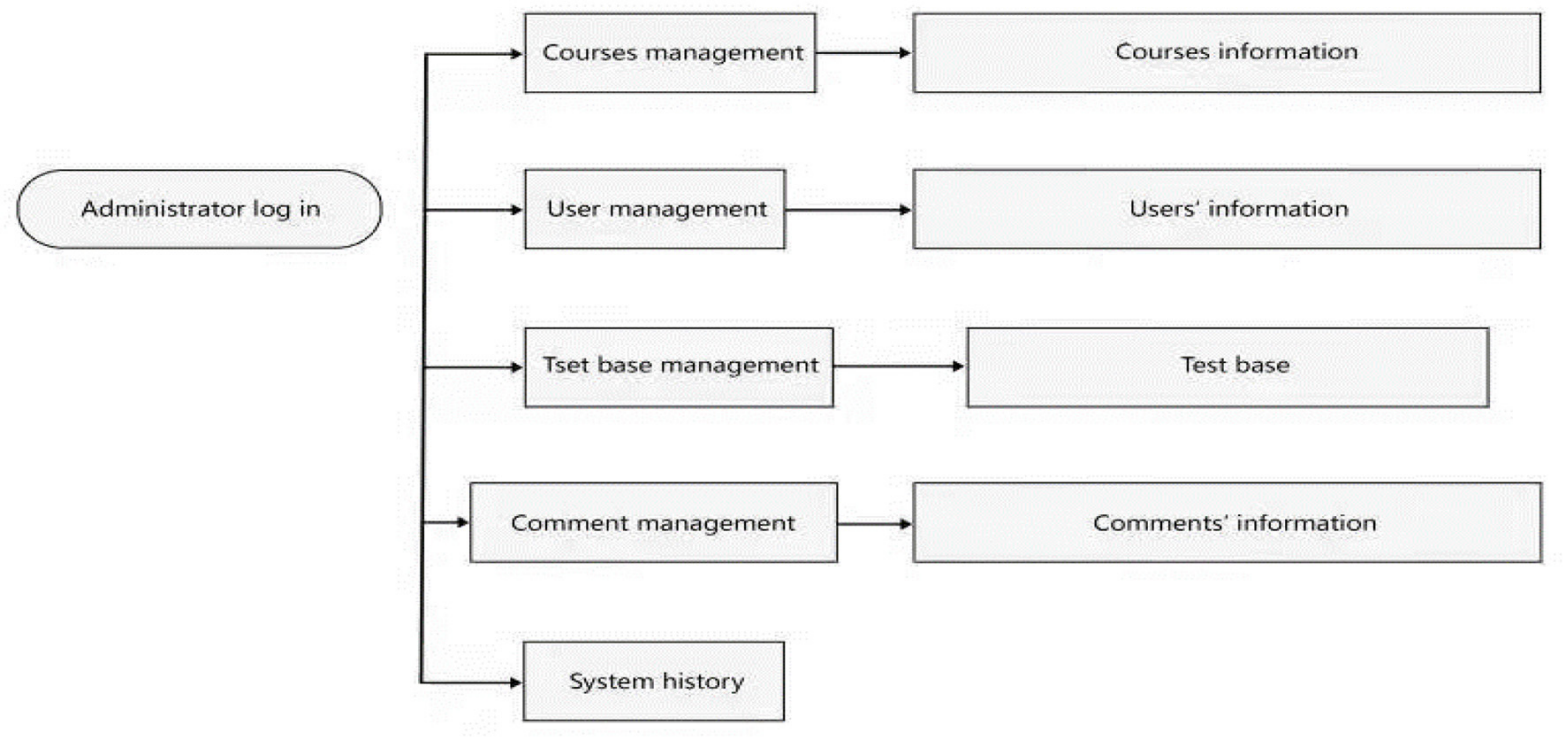
Flowchart of the background management mode.

After users log in to the BBS, they can view the posts of other users in the article reading area and recommendation discussion area, in which they can also search for their interested posts according to keywords. Under the post, their comments can be deleted and edited, users can also post a comment under their own post. The administrator has the right to supervise the posts of the forum. For example, good posts should be highlighted, disruptive posts needed to be deleted. These behaviors are aimed at the operation of the post and designed to manage the post module. The general operation flow of the background management module is shown in Figure 4.

After logging in, the administrator can add, delete, and check the four tables: The course information, the user information, the question library, and comment information. The system records the number of logins on the same day, the number of active users on the same day, the current number of online users, etc.

### Analysis of the Use Cases of the Guangfu Cultural Analysis Online Education Platform

**Figure 5:**
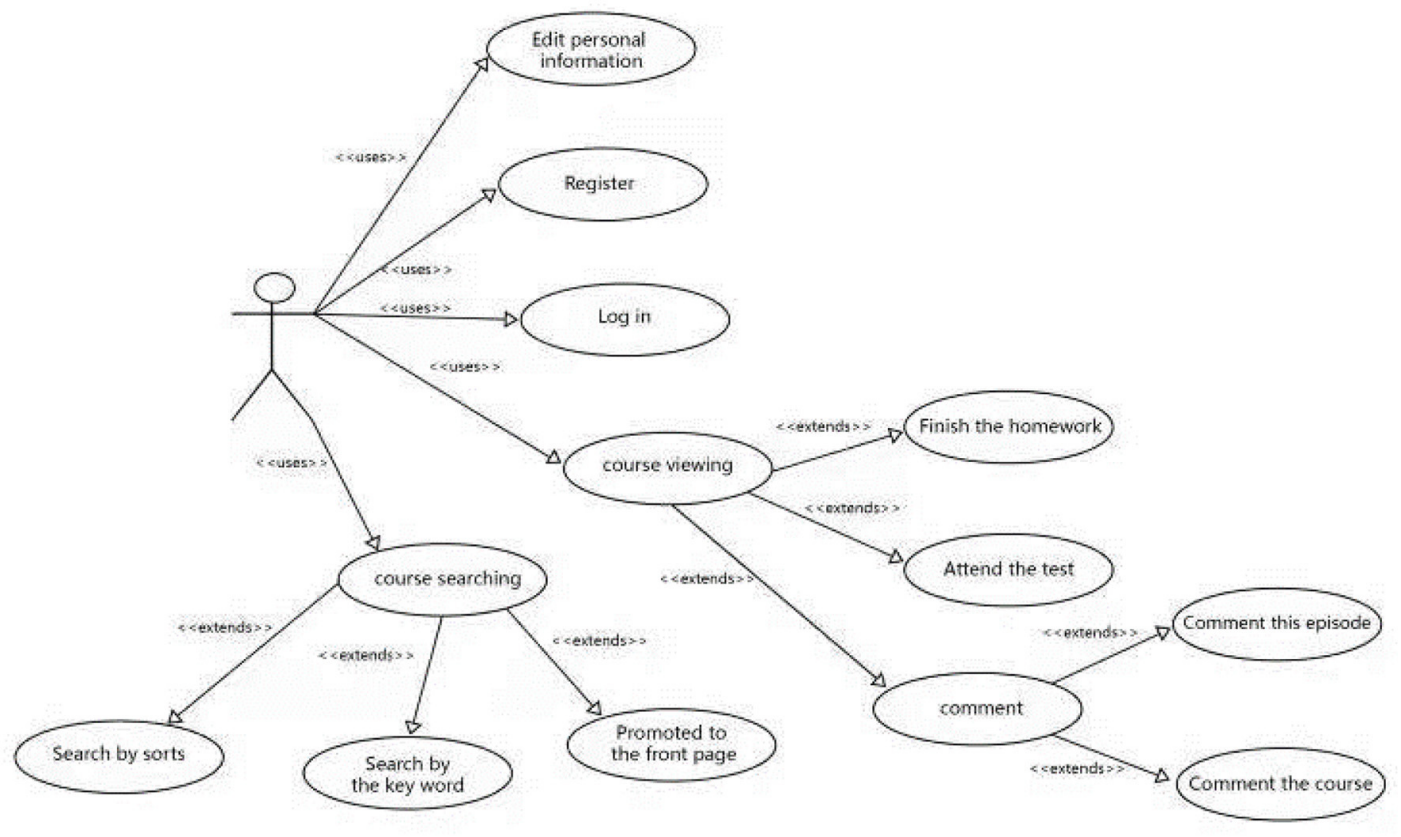
Operating flow of the ordinary user.

The first use case is of the user under the operating module, which is divided into two permission roles: The teacher, as the course publisher and the ordinary user. The use case diagram of the ordinary user is shown in Table 1 and Figure 5.

**Table 1:** User operation cases.

Use Case Name: User Operation Use Case
Use case ID number: 001
Participants: General user
Brief description: Users search for courses, watch courses, register, sign in, and edit personal information
Pre-conditions: User account password is correct; permission is ordinary user
Basic event flow:
1. User login
2. User registration
3. Users manage their personal information and edit their own information
4. User search course via category search, search by keyword, the homepage recommendation
5. Users watch the course
6. Users watch the course (after completing their homework, exams, comments)
Other event stream: The user logs off the current login
Exception event flow: Program error, return to the home page
Post-conditions:
Note:

The use case diagram of the teacher's course publisher permission is shown in Table 2.

**Table 2:** Administrator operation use cases.

Use case name: Administrator action use case
Use case ID number: 003
Participants: The administrator
Brief description: Administrator management course, management users, management question library, management forum post
Pre-conditions: User account password is correct, permission is administrator
Basic event flow:
1. The administrator management course
1.1 Recommended on the management home page
1.2 Increase and deletion of the management curriculum list
2. The administrator manages the addition and deletion of the user table.
3. The administrator manages the addition and deletion of the question database
4. Administrator management forum post
4.1 Recommended on the management home page
4.2 Management of the addition and deletion of posts and post forms
Other event stream: Administrator cancels login
Exception event flow: Program error message, returns to the home page
Post-conditions:
Comment: The administrator has the highest system permissions to operate other permissions roles

## Implementation and Testing

### Code Realization of the Guangfu Cultural Online Education Platform

#### Video Playback Code Implementation

For an online education platform, course video playing is an important teaching strategy. The development of the Guangfu Culture online education platform applied the JMF standard, launched by Java to realize the video playback function. The advantages and disadvantages of the JMF components are obvious. This component is versatile, while its visual effect is average. Due to the limited technical level, third-party video playing components could not be used here to further illustrate this function. During the development process, many difficulties were encountered, one of which was how to verify the user's mobile phone number at registration, that is, to realize the SMS sending function. A lot of information was gathered, after which an API interface was provided by the operator to complete this effect.

#### User Login Filter Implementation

Filters can filter the user's login request. In this platform, if the user did not log in and view the JSP page directly, the system gave prompts and jumped to the landing page.

**Figure 6:**
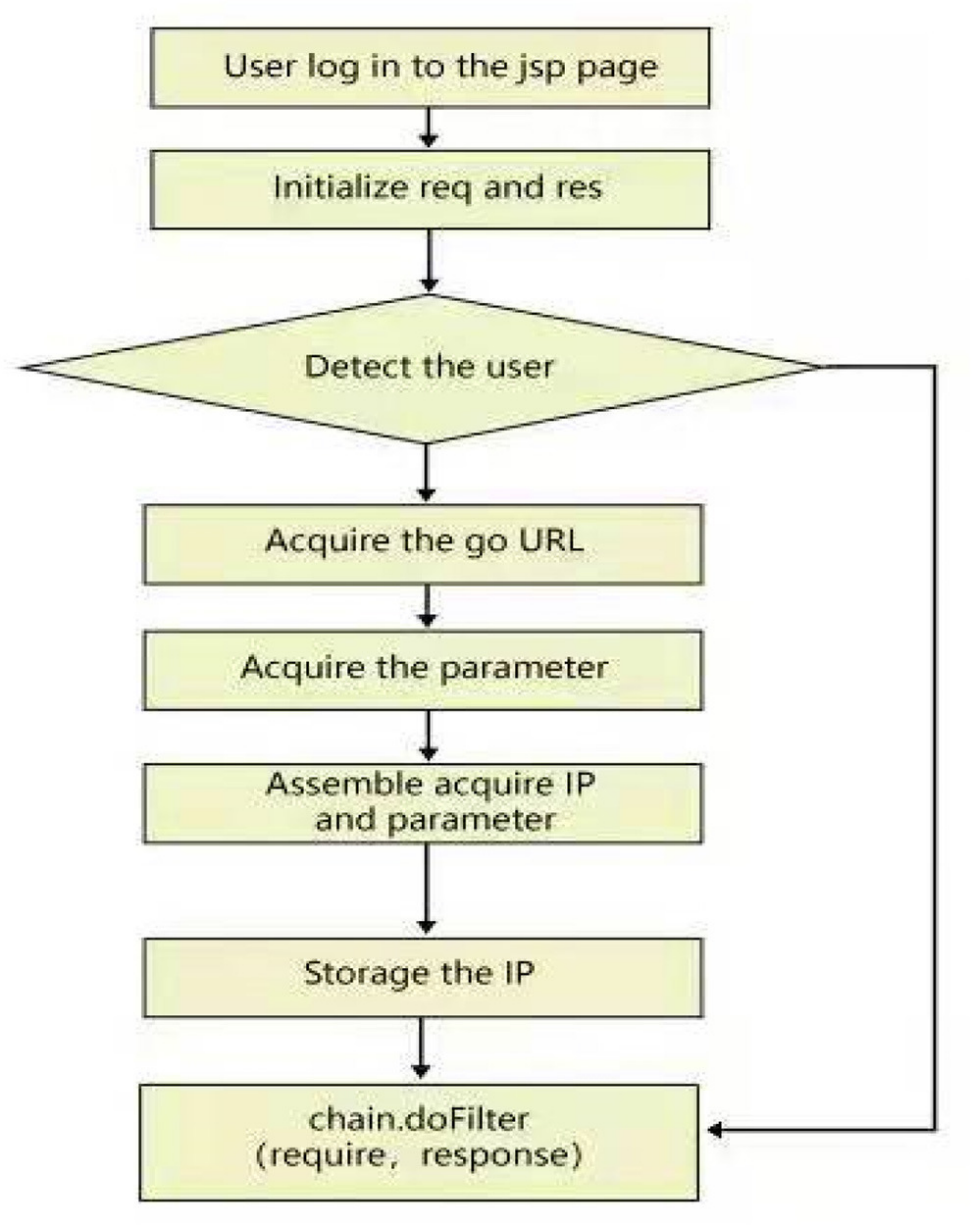
The SSH framework.

In addition to the SSH framework, C3P0 data sources, AJAX, and many third-party interfaces were used in the development process (this code is not displayed). The flowchart of the program is shown in Figure 6.

### Online Education and Artificial Intelligence Platform Testing

After completing the development, the Black Box test method was used to comprehensively test the functions of the whole platform. The following two functions of posting and posting in the forum module were used as examples. Manual operation and using the idea of a causal map in the Black Box test mainly tested whether the function of the target platform could meet the requirements or not. A total of 2,065 students in school took the test and more than 3,000 other samples outside the school were also included.

#### Test Forum Posting and the Post Function

After testing, the post posting function of the forum module is normal and can be put into use as shown in Table 3.

**Table 3:** Test forum comments and posting functions.

**Reason**	**Expected results**	**Actual results**	**Test results**
Posting when not logged in	Please Log in	Please Log in	Success
The post title is empty	Pops “title can't be empty”	Pops “title can't be empty”	Success
The posting content is empty	Popup “Content cannot be empty”	Popup “Content cannot be empty”	Success
Posts did not select the classification	Use the default classification	Use the default classification	Success
Post the verification code filling in the error	Popup the verification code error	Popup the verification code error	Success
Post poster when not logged in	Please Log in	Please Log in	Success
The post content is empty	Popup “Content cannot be empty”	Popup “Content cannot be empty”	Success

#### Test Feedback and Limitation

The system ran through a series of tests before the official opening; a few bugs were detected in the testing process and the error rate was under 0.1%. Most of the errors emerged in the interactive comment summiting process, the sensitive words shielding setting might hamper students from summiting if such words are used, even if sentences are made from such words. During the official running, 2,065 students in our school and more than 3,000 other samples outside the school participated in the official test, the favorable rate was 85%. More than 12,000 people outside the systems also read about our sources. Our system has proven its high efficiency via the test, but it is still confined to the current website learning mode. The teacher-student communications still deeply relied on wires and the device will greatly influence the knowledge imparting process. Our system's operation has great reliance on website stability, there is no backup operation platform for our system.

## Conclusion

In today's society, material consumption has greatly increased compared to 20 years ago, so the spiritual level should be elevated as well. Realizing the “Chinese dream” is an ongoing challenge, but if Chinese people leave out traditional culture and forget their roots in this pursuit, then they are no longer who they are. The spread of traditional culture should become an industry, to form a pattern. Traditional culture should not be seen simply as a research subject, there should be experts and scholars from different fields to participate in traditional culture modernization via the communication industry. On the other hand, every great progress in various research fields has come from something that has not yet been explored, especially for the development of “Internet +.” Today, in the electronic industry, everyone is looking for Internet development from an undeveloped aspect, trying to occupy the unknown market. The spread of traditional culture through the Internet is undoubtedly a good opportunity, once there is a successful case, various firms will be encouraged to engage. By that time, the spread and development of traditional culture will certainly enter a golden age. This platform contribution has been proven to be effective in the 5,056 samples in traditional culture education, and as the Internet is developing faster than before, it will provide a larger user base. The users in the platform have a longer study time, compared with non-platform users, and can mesmerize traditional culture knowledge better than non-users (21.5% higher in the performance test). The performance of students and the platform's efficiency have great reliance on the devices.

In a word, to have a clear vision for the online education industry, we should summarize the characteristics of Guangfu culture and design a suitable set of curriculum framework and teaching systems for “Guangfu culture.” In future work, teacher professional knowledge upgrades are particularly important in order for them to cooperate with government policies, media guidance, university research direction, We-Media propaganda, etc. To attract society's interest for Guangfu culture, we need to recognize its importance and attract people's interest in learning it.

The 21st century is an era of information explosion, and it is also an era of knowledge economy. With social progression and development, there is increasing demand for knowledge and people should accept different new things, such as traditional culture. As the saying goes, “to live and to learn” is getting more realistic than any previous era. Lifelong education has become a representative characteristic of this era, and more and more people tend to obtain their knowledge from the network, yet it is hard to distinguish a really practical platform. The impact of online education on individual education environments will have the following characteristics: First, the concept of a global village, explosive information, and global resources sharing driven by the Internet have become prevalent. Second, the performance mode of curriculum design is more and more diverse. Education modes have moved from reality to virtual. Third, students' learning mode is transforming to self-supervision and self-discipline learning. The interaction between the online educator and learners is mainly through the Internet, from which they can find the knowledge information that they need. Nowadays, with the continuous development of information technology, network teaching is an inevitable practice. It is a good medium and carrier for the popularization, democratization, and longevity of education.

Now, by smoothing out the ideas, the inheritance and innovation of the “Guangfu Culture” should be organically combined with new media. Even if there are some drawbacks, it is inevitable for the traditional culture to meet the needs of the developing times and realize the transformation of the times. The modern transformation and innovation of Guangfu culture needs to be jointly promoted by experts and scholars with a strong sense of responsibility. The culture is of one soil and soil breeds one side, and one culture affects the economy of one side and creates talents of one side. Guangfu culture is the root of the Pearl River Delta region, it is the emotional support of Cantonese people in today's rapid economic development environment, it might benefit social and economic aspects greatly.

It is unforgivable for us to forget our tradition. Missing the tradition is missing the spirit. Lacking the Guangfu culture is missing the “Cantonese spirit.” Therefore, the development of modern Internet technology and convenient and fast communication is actually an opportunity for us to create a good platform for traditional regional culture to thrive in this modern age.

## Author Contributions

The author confirms being the sole contributor of this work and has approved it for publication.

## Conflict of Interest

The author declares that the research was conducted in the absence of any commercial or financial relationships that could be construed as a potential conflict of interest.

## Publisher's Note

All claims expressed in this article are solely those of the authors and do not necessarily represent those of their affiliated organizations, or those of the publisher, the editors and the reviewers. Any product that may be evaluated in this article, or claim that may be made by its manufacturer, is not guaranteed or endorsed by the publisher.
